# Temperature and Concentration Stratification Effects in Mixed Convection Flow of an Oldroyd-B Fluid with Thermal Radiation and Chemical Reaction

**DOI:** 10.1371/journal.pone.0127646

**Published:** 2015-06-23

**Authors:** Tasawar Hayat, Taseer Muhammad, Sabir Ali Shehzad, Ahmed Alsaedi

**Affiliations:** 1 Department of Mathematics, Quaid-I-Azam University, 45320, Islamabad, 44000, Pakistan; 2 Nonlinear Analysis and Applied Mathematics (NAAM) Research Group, King Abdulaziz University, P. O. Box 80203, Jeddah, 21589, Saudi Arabia; 3 Department of Mathematics, Comsats Institute of Information Technology, Sahiwal, 57000, Pakistan; Bascom Palmer Eye Institute, University of Miami School of Medicine, UNITED STATES

## Abstract

This research addresses the mixed convection flow of an Oldroyd-B fluid in a doubly stratified surface. Both temperature and concentration stratification effects are considered. Thermal radiation and chemical reaction effects are accounted. The governing nonlinear boundary layer equations are converted to coupled nonlinear ordinary differential equations using appropriate transformations. Resulting nonlinear systems are solved for the convergent series solutions. Graphs are plotted to examine the impacts of physical parameters on the non-dimensional temperature and concentration distributions. The local Nusselt number and the local Sherwood number are computed and analyzed numerically.

## Introduction

Analysis of non-Newtonian fluids has great importance due to its several industrial and engineering applications. In particular these fluids are encountered in the material processing, chemical and nuclear industries, bioengineering, oil reservoir engineering, polymeric liquids and foodstuffs. Several fluids like paints, paper pulp, shampoos, ketchup, apple sauce, slurries, certain oils and polymer solutions are the non-Newtonian fluids. The characteristics of all the non-Newtonian fluids cannot be explained via one constitutive relationship. Hence various fluid models are proposed in the literature for the properties of non-Newtonian fluids. Generally non-Newtonian materials are classified under three categories namely (i) differential type (ii) rate type and (iii) integral type. The Maxwell fluid model is the simplest subclass of rate type fluids. This model describes only the properties of relaxation time. The characteristics of retardation time cannot be predicted by the Maxwell fluid. An Oldroyd-B fluid model was developed to examine both the relaxation and retardation times characteristics. Instabilities in viscoelastic liquids were studied by Larson [1]. In this investigation, he discussed the instabilities in Taylor-Couette flows, instabilities in cone-and-plate and plate-and-plate flows, instabilities in parallel shear flows, instabilities in external and multi-dimensional flows. The instabilities in the flows occurring in the absence of inertial forces were investigated by Shaqfeh [2]. Laso and Ottinger [3] presented a study to examine the numerical simulation of viscoelastic liquids based on molecular models. Rajagopal and Bhatnagar [4] computed the asymptotically decaying solution of an Oldroyd-B fluid past an infinite porous plate. Thermodynamic properties of rate type non-Newtonian fluids were investigated by Rajagopal and Srinivasa [5]. Numerical solutions of Oldroyd-B and PTT-fluids with both the linear and exponential stress functions were developed by Alves et al. [6]. Some recent investigations on non-Newtonian fluids can be seen in the references [7–14].

Heat and mass transfer analysis in the boundary layer flow over a stretching surface has key role in the industrial and engineering applications, for example, manufacturing of plastic and rubber sheets, annealing and thinning of copper wires, drawing on stretching sheets through quiescent fluids, boundary layer along a liquid film condensation process, damage of crops due to freezing, desalination, refrigeration and air conditioning, compact heat exchangers, solar power collectors, human transpiration and many others (see refs. [15,16]). Heat and mass transfer effects in boundary layer flow of viscoelastic fluid with thermal slip condition were investigated by Turkyilmazoglu [17]. Hayat and Alsaedi [18] carried out a study to examine the heat and mass transfer phenomena in buoyancy driven flow of an Oldroyd-B fluid. Thermophoresis and Joule heating effects are further considered. Hayat et al. [19] presented the series solutions of magnetohydrodynamic (MHD) flow of Casson fluid with heat and mass transfer. Soret and Dufour effects are present in this investigation. Mixed convection flow of Jeffrey fluid in the presence of heat and mass transfer is investigated by Shehzad et al. [20]. Gupta et al. [21] discussed the effect of cadmium on growth and active constituents of bacopa monnieri. Induced magnetic field effect in mixed convection peristaltic flow of third order fluid with nanoparticles is discussed by Noreen [22]. Bachok et al. [23] studied the boundary layer flow of viscous fluid in presence of mixed convection and viscous dissipation. Su et al. [24] developed a lattice Boltzmann method coupled with the Oldroyd-B constitutive equation to stimulate flow of viscoelastic fluid. Here the numerical results of 2D channel flow agree well with the analytical and some experimental results reported in the previous studies. Slip effects in peristaltic flow of generalised Oldroyd-B fluids is explored by Tripathi et al. [25]. They computed the homotopic solutions of the modelled differential system.

Effect of stratification is an important aspect in heat and mass transfer analyses. Stratification of fluids occurs due to temperature variations, concentration differences or the presence of different fluids of different densities. When the heat and mass transfer are present simultaneously then it is important to analyze the effect of double stratification on the convective flows. The analysis of mixed convection in a doubly stratified medium is an important problem. It is because of its occurrence in geophysical flows (see ref. [26]). Such flows involve in the rivers, lakes and seas, thermal energy storage systems and solar ponds etc. Chang and Lee [27] investigated the free convection flow by a vertical plate with uniform and constant heat flux in a thermally stratified micropolar fluid. Cheng [28] examined the combined heat and mass transfer effect in natural convection flow from a vertical wavy surface in a power-law fluid saturated porous medium. Both thermal and mass stratification effects were present. Srinivasacharya and Reddy [29] discussed the effect of double stratification in mixed convection flow of micropolar fluid. Effect of double stratification on MHD free convection flow of micropolar fluid is investigated by Srinivasacharya and Upendar [30]. Non-Darcy mixed convection flow in a doubly stratified medium under Soret and Dufour effects is studied by Srinivasacharya and Surender [31]. Srinivasacharya and Surender [32] addressed the effect of double stratification on mixed convection boundary layer flow of a nanofluid past a vertical plate in porous medium.

The basic theme of present study is to investigate the effects of thermal radiation, chemical reaction, thermal and solutal stratification in the mixed convection boundary layer flow of an Oldroyd-B fluid over a stretching surface. The studies available in the literature on this topic mostly dealt with the thermal stratification effect. Some recent aforementioned studies investigated the effects of both thermal and concentration stratification in viscous fluid flow. This is the first attempt to study such effects for non-Newtonian fluids. Mathematical modelling is developed under the consideration of thermal and concentration stratification effects. The series solutions to the resulting nonlinear differential systems are constructed via homotopy analysis method (HAM) [33–41]. The effects of various emerging parameters on the temperature and concentration fields are presented through plots and tables. The local Nusselt and the local Sherwood numbers are computed numerically and analyzed.

## Mathematical Modeling

We consider the steady two-dimensional doubly stratified mixed convection flow of an incompressible Oldroyd-B fluid. The flow is caused by a linearly stretching surface at *y* = 0. The flow occupies the domain *y* > 0. Boundary layer flow is considered in the presence of thermal radiation and first order chemical reaction. The governing boundary layer equations for incompressible flow of an Oldroyd-B fluid with heat and mass transfer are given below (see Appendix for detailed derivation):

$$\frac{\partial u}{\partial x}+\frac{\partial v}{\partial y}=0,$$ (1)

$$\begin{array}{r} u\frac{\partial u}{\partial x}+v\frac{\partial u}{\partial y}+\lambda_{1}\left(\begin{array}{c} u^{2}\frac{\partial^{2}u}{\partial x^{2}}+v^{2}\frac{\partial^{2}u}{\partial y^{2}}\\ +2uv\frac{\partial^{2}u}{\partial x\partial y} \end{array}\right)=\nu \frac{\partial^{2}u}{\partial y^{2}}+\nu \lambda_{2}\left(\begin{array}{c} u\frac{\partial^{3}u}{\partial x\partial y^{2}}+v\frac{\partial^{3}u}{\partial y^{3}}\\ -\frac{\partial u}{\partial x}\frac{\partial^{2}u}{\partial y^{2}}-\frac{\partial u}{\partial y}\frac{\partial^{2}v}{\partial y^{2}} \end{array}\right)\;\\ +g\left(\beta_{T}\left(T-T_{\infty }\right)+\beta_{C}\left(C-C_{\infty }\right)\right), \end{array}$$ (2)

$$\frac{1}{\rho }\frac{\partial p}{\partial y}=-\frac{\lambda_{1}}{\rho }\left(-\frac{\partial v}{\partial y}\frac{\partial p}{\partial x}+u\frac{\partial^{2}p}{\partial x\partial y}\right),$$ (3)

$$u\frac{\partial T}{\partial x}+v\frac{\partial T}{\partial y}=\alpha \frac{\partial^{2}T}{\partial y^{2}}-\frac{1}{\rho c_{p}}\frac{\partial q_{r}}{\partial y},$$ (4)

$$u\frac{\partial C}{\partial x}+v\frac{\partial C}{\partial y}=D\frac{\partial^{2}C}{\partial y^{2}}-K_{1}\left(C-C_{\infty }\right).$$ (5)

The appropriate boundary conditions are

$$u=U_{w}\left(x\right),\;v=0,\;T=T_{w}\left(x\right),\;C=C_{w}\left(x\right)\;\text{at}\;y=0,$$ (6)

$$u\to 0,\;T\to T_{\infty }\left(x\right)=T_{\infty ,\;0}+A_{1}x^{2},\;C\to C_{\infty }\left(x\right)=C_{\infty ,\;0}+B_{1}x^{2}\;\text{as}\;y\to \infty ,$$ (7)

where *u* and *v* are the velocity components in the *x*– and *y*–directions respectively, *λ*_1_ the relaxation time, *ν* = *μ* / *ρ* the kinematic viscosity, *μ* the dynamic viscosity, *ρ* the density of fluid, *λ*_2_ the retardation time, *g* the gravitational acceleration, *β*_*T*_ the thermal expansion coefficient, *T* the temperature, *β*_*C*_ the concentration expansion coefficient, *C* the concentration, *α* = *k* / *ρc*_*p*_ the thermal diffusivity of the fluid, *k* the thermal conductivity, *c*_*p*_ the specific heat at constant pressure, *q*_*r*_ the radiative heat flux, *D* the diffusion coefficient, *K*_1_ the reaction rate, *T*_*w*_ and *T*_*∞*_ the temperatures of the surface and far away from the surface and *C*_*w*_ and *C*_*∞*_ the concentrations at the surface and far away from the surface. The subscript *w* denotes the wall condition. This study assumes that the surface stretching velocity, wall temperature and wall concentration are

$$U_{w}\left(x\right)=ax,\;T_{w}\left(x\right)=T_{\infty ,\;0}+M_{1}x^{2},\;C_{w}\left(x\right)=C_{\infty ,\;0}+N_{1}x^{2}.$$ (8)

where *a*, *A*_1_, *B*_1_, *M*_1_, *N*_1_, *T*_∞,0_ and *C*_∞,0_ are the positive constants. The radiative heat flux *q*_*r*_ via Rosseland's approximation can be expressed as follows:

$$q_{r}=-\frac{4\sigma_{1}}{3m}\frac{\partial \left(T^{4}\right)}{\partial y},$$ (9)

in which *σ*_1_ is the Stefan-Boltzman constant and *m* is the mean absorption coefficient. We assume that the difference in temperature within the flow is such that *T* ^4^ can be written as a linear combination of temperature. By employing Taylor's series and neglecting higher order terms we have [18]:

$$T^{4}≅-3T_{\infty }^{4}+4T_{\infty }^{3}T,$$ (10)

Substituting Eq (10) in Eq (9) we get

$$\frac{\partial q_{r}}{\partial y}=-\frac{16\sigma_{1}T_{\infty }^{3}}{3m}\frac{\partial^{2}T}{\partial y^{2}},$$ (11)

Using Eq (11) in Eq (4) we have

$$u\frac{\partial T}{\partial x}+v\frac{\partial T}{\partial y}=\alpha \frac{\partial^{2}T}{\partial y^{2}}+\frac{16\sigma_{1}T_{\infty }^{3}}{3m\rho c_{p}}\frac{\partial^{2}T}{\partial y^{2}}.$$ (12)

The dimensionless variables can be defined as follows:

$$\begin{array}{c} u=axf\prime \left(\eta \right),\;v=-\sqrt{a\nu }f\left(\eta \right),\;\eta =\sqrt{\frac{a}{\nu }}y,p=\frac{\mu }{x}U_{w}P\left(\eta \right),\\ \theta \left(\eta \right)=\frac{T-T_{\infty ,\;0}}{\Delta T}-\frac{A_{1}x^{2}}{\Delta T},\;\Delta T=T_{w}\left(x\right)-T_{\infty ,\;0}=M_{1}x^{2},\\ \phi \left(\eta \right)=\frac{C-C_{\infty ,\;0}}{\Delta C}-\frac{B_{1}x^{2}}{\Delta C},\;\Delta C=C_{w}\left(x\right)-C_{\infty ,\;0}=N_{1}x^{2}, \end{array}\}$$ (13)

Incompressibility condition is now identically satisfied and Eqs (2)–(8) and (12) become

$$f\prime \prime \prime +ff\prime \prime -f\prime^{2}+\beta_{1}\left(2ff\prime f\prime \prime -f^{2}f\prime \prime \prime \right)+\beta_{2}\left(f\prime \prime^{2}-ff^{iv}\right)+\lambda \left(\theta +N\phi \right)=0,$$ (14)

P'=0, (15)

$$\left(1+Rd\right)\theta \prime \prime +\text{Pr}\left(f\theta \prime -2f\prime \theta -2\varepsilon_{1}f\prime \right)=0,$$ (16)

$$\phi \prime \prime +Sc\left(f\phi \prime -2f\prime \phi -\gamma \phi -2\varepsilon_{2}f\prime \right)=0,$$ (17)

$$f=0,\;f\prime =1,\;\theta =1-\varepsilon_{1},\;\phi =1-\varepsilon_{2}\;\text{at}\;\eta =0,$$ (18)

f′→0,θ→0,ϕ→0asη→∞. (19)

In above expressions *β*_1_ and *β*_2_ are the Deborah numbers in terms of relaxation and retardation times respectively, *λ* is the mixed convection parameter, *Gr*_*x*_ is the Grashof number, Re_*x*_ is the local Reynolds number, *N* is the buoyancy ratio parameter, *Rd* is the thermal radiation parameter, Pr is the Prandtl number, *ε*_1_ is the thermal stratification parameter, *Sc* is the Schmidt number, *γ* is the chemical reaction parameter, *ε*_2_ is the solutal stratification parameter and prime stands for differentiation with respect to *η*. Note that when *β*_2_ = *0*, this analysis reduced to the Maxwell fluid flow case. Eq (15) indicates that *P* is constant in the *y*–direction. The involved variables can be defined as follows:

$$\begin{array}{c} \beta_{1}=\lambda_{1}a,\;\beta_{2}=\lambda_{2}a,\;\lambda =\frac{Gr_{x}}{{\text{Re}}_{x}^{2}},\;Gr_{x}=\frac{g\beta_{T}\Delta Tx^{3}}{\nu^{2}},\\ {\text{Re}}_{x}=\frac{U_{w}x}{\nu },\;N=\frac{\beta_{C}\Delta C}{\beta_{T}\Delta T},\;Rd=\frac{16\sigma_{1}T_{\infty }^{3}}{3km},\;\text{Pr}=\frac{\nu }{\alpha },\\ \varepsilon_{1}=\frac{x}{\Delta T}\frac{d}{dx}\left[T_{\infty }\left(x\right)\right],\;Sc=\frac{\nu }{D},\;\gamma =\frac{K_{1}}{a},\;\varepsilon_{2}=\frac{x}{\Delta C}\frac{d}{dx}\left[C_{\infty }\left(x\right)\right]. \end{array}\}$$ (20)

The local Nusselt number *Nu*_*x*_ and the local Sherwood number *Sh*_*x*_ are given by

$$Nu_{x}=-\frac{x\left(k+\frac{16\sigma_{1}T_{\infty }^{3}}{3m}\right)}{k\left(T_{w}-T_{\infty }\right)}{\frac{\partial T}{\partial y}|}_{y=0}=-{\left({\text{Re}}_{x}\right)}^{1/2}\left(1+Rd\right)\theta \prime \left(0\right),$$ (21)

$$Sh_{x}=-\frac{x}{\left(C_{w}-C_{\infty }\right)}{\frac{\partial C}{\partial y}|}_{y=0}=-{\left({\text{Re}}_{x}\right)}^{1/2}\phi \prime \left(0\right).$$ (22)

## Series Solutions

The initial guesses and the linear operators are

$$f_{0}(\eta )=1-e^{-\eta },\;\theta_{0}(\eta )=\left(1-\varepsilon_{1}\right)e^{-\eta },\;\phi_{0}(\eta )=\left(1-\varepsilon_{2}\right)e^{-\eta },$$ (23)

$$L_{f}=f\prime \prime \prime -f\prime ,\;L_{\theta }=\theta \prime \prime -\theta ,\;L_{\phi }=\phi \prime \prime -\phi .$$ (24)

The operators satisfy the following properties [33]:

$$L_{f}\left[C_{1}+C_{2}e^{\eta }+C_{3}e^{-\eta }\right]=0,\;L_{\theta }\left[C_{4}e^{\eta }+C_{5}e^{-\eta }\right]=0,\;L_{\phi }\left[C_{6}e^{\eta }+C_{7}e^{-\eta }\right]=0,$$ (25)

in which *C*_*i*_ (*i* = 1–7) are the arbitrary constants.

We can define the following zeroth-order deformation problems [36,40]:

$$(1-p)L_{f}\left[\hat{f}(\eta ,p)-f_{0}(\eta )\right]=p\hbar_{f}N_{f}[\hat{f}(\eta ,p),\hat{\theta }(\eta ,p),\hat{\phi }(\eta ,p)],$$ (26)

$$(1-p)L_{\theta }\left[\hat{\theta }(\eta ,p)-\theta_{0}(\eta )\right]=p\hbar_{\theta }N_{\theta }[\hat{f}(\eta ,p),\hat{\theta }(\eta ,p),\hat{\phi }(\eta ,p)],$$ (27)

$$(1-p)L_{\phi }\left[\hat{\phi }(\eta ,p)-\phi_{0}(\eta )\right]=p\hbar_{\phi }N_{\phi }[\hat{f}(\eta ,p),\hat{\theta }(\eta ,p),\hat{\phi }(\eta ,p)],$$ (28)

$$\hat{f}(0,p)=0,\;\hat{f}\prime (0,p)=1,\;\hat{\theta }(0,p)=1-\varepsilon_{1},\;\hat{\phi }(0,p)=1-\varepsilon_{2},$$ (29)

$$\hat{f}\prime (\infty ,p)=0,\;\hat{\theta }(\infty ,p)=0,\;\hat{\phi }(\infty ,p)=0,$$ (30)

$$\begin{array}{c} N_{f}\left[\hat{f}(\eta ;p),\hat{\theta }(\eta ,p),\hat{\phi }(\eta ,p)\right]=\frac{\partial^{3}\hat{f}}{\partial \eta^{3}}+\hat{f}\frac{\partial^{2}\hat{f}}{\partial \eta^{2}}-{\left(\frac{\partial \hat{f}}{\partial \eta }\right)}^{2}+\beta_{1}\left(2\hat{f}\frac{\partial \hat{f}}{\partial \eta }\frac{\partial^{2}\hat{f}}{\partial \eta^{2}}-{\hat{f}}^{2}\frac{\partial^{3}\hat{f}}{\partial \eta^{3}}\right)\\ \;+\beta_{2}\left({\left(\frac{\partial^{2}\hat{f}}{\partial \eta^{2}}\right)}^{2}-\hat{f}\frac{\partial^{4}\hat{f}}{\partial \eta^{4}}\right)+\lambda \left(\hat{\theta }+N\hat{\phi }\right), \end{array}$$ (31)

$$N_{\theta }\left[\hat{f}(\eta ;p),\hat{\theta }(\eta ,p),\hat{\phi }(\eta ,p)\right]=\left(1+Rd\right)\frac{\partial^{2}\hat{\theta }}{\partial \eta^{2}}+\text{Pr}\left(\hat{f}\frac{\partial \hat{\theta }}{\partial \eta }-2\frac{\partial \hat{f}}{\partial \eta }\hat{\theta }-2\varepsilon_{1}\frac{\partial \hat{f}}{\partial \eta }\right),$$ (32)

$$N_{\phi }[\hat{f}(\eta ;p),\hat{\theta }(\eta ,p),\hat{\phi }(\eta ,p)]=\frac{\partial^{2}\hat{\phi }}{\partial \eta^{2}}+Sc\left(\hat{f}\frac{\partial \hat{\phi }}{\partial \eta }-2\frac{\partial \hat{f}}{\partial \eta }\hat{\phi }-\gamma \hat{\phi }-2\varepsilon_{2}\frac{\partial \hat{f}}{\partial \eta }\right).$$ (33)

Here *p* denotes the embedding parameter $\hbar_{f},$ $\hbar_{\theta }$ and $\hbar_{\phi }$ the non-zero auxiliary parameters and **N**_*f*_, **N**_*θ*_ and **N**_*ϕ*_ the nonlinear operators. Setting *p* = 0 and *p* = 1 we have [35,38]:

$$\hat{f}(\eta ;0)=f_{0}(\eta ),\;\hat{f}(\eta ;1)=f(\eta ),$$ (34)

$$\hat{\theta }(\eta ,0)=\theta_{0}(\eta ),\;\hat{\theta }(\eta ,1)=\theta (\eta ),$$ (35)

$$\hat{\phi }(\eta ,0)=\phi_{0}(\eta ),\;\hat{\phi }(\eta ,1)=\phi (\eta ).$$ (36)

When *p* varies from 0 to 1 then $\hat{f}(\eta ;p),$ $\hat{\theta }(\eta ,p)$ and $\hat{\phi }(\eta ,p)$ vary from the initial guesses *f*_0_(*η*), *θ*_0_(*η*) and *ϕ*_0_(*η*) to the final solutions *f*(*η*), *θ*(*η*) and *ϕ*(*η*), respectively. Taylor series expansion gives [37,39]:

$$\hat{f}(\eta ;p)=f_{0}(\eta )+\overset{\infty }{\underset{m=1}{\sum }}f_{m}(\eta )p^{m},\;f_{m}(\eta )={\frac{1}{m!}\frac{\partial^{m}\hat{f}(\eta ,p)}{\partial p^{m}}|}_{p=0},$$ (37)

$$\hat{\theta }(\eta ,p)=\theta_{0}(\eta )+\overset{\infty }{\underset{m=1}{\sum }}\theta_{m}(\eta )p^{m},\;\theta_{m}(\eta )={\frac{1}{m!}\frac{\partial^{m}\hat{\theta }(\eta ,p)}{\partial p^{m}}|}_{p=0},$$ (38)

$$\hat{\phi }(\eta ,p)=\phi_{0}(\eta )+\overset{\infty }{\underset{m=1}{\sum }}\phi_{m}(\eta )p^{m},\;\phi_{m}(\eta )={\frac{1}{m!}\frac{\partial^{m}\hat{\phi }(\eta ,p)}{\partial p^{m}}|}_{p=0}.$$ (39)

The convergence of above series strongly depends upon $\hbar_{f},$ $\hbar_{\theta }$ and $\hbar_{\phi }.$ Considering that $\hbar_{f},$ $\hbar_{\theta }$ and $\hbar_{\phi }$ are chosen in such a manner that Eqs (37)–(39) converge at *p* = 1 then [33,34]:

$$\hat{f}(\eta ;p)=f_{0}(\eta )+\overset{\infty }{\underset{m=1}{\sum }}f_{m}(\eta ),$$ (40)

$$\hat{\theta }(\eta ,p)=\theta_{0}(\eta )+\overset{\infty }{\underset{m=1}{\sum }}\theta_{m}(\eta ),$$ (41)

$$\hat{\phi }(\eta ,p)=\phi_{0}(\eta )+\overset{\infty }{\underset{m=1}{\sum }}\phi_{m}(\eta ).$$ (42)

The mth-order problems are [33]:

$$L_{f}\left[f_{m}(\eta )-\chi_{m}f_{m-1}(\eta )\right]=\hbar_{f}R_{f}^{m}(\eta ),$$ (43)

$$L_{\theta }\left[\theta_{m}(\eta )-\chi_{m}\theta_{m-1}(\eta )\right]=\hbar_{\theta }R_{\theta }^{m}(\eta ),$$ (44)

$$L_{\phi }\left[\phi_{m}(\eta )-\chi_{m}\phi_{m-1}(\eta )\right]=\hbar_{\phi }R_{\phi }^{m}(\eta ),$$ (45)

$$f_{m}(0)=f_{m}^{\prime }(0)=f_{m}^{\prime }(\infty )=0,$$ (46)

$$\theta_{m}(0)=\theta_{m}(\infty )=0,\;\phi_{m}^{\prime }(0)=\phi_{m}(\infty )=0,$$ (47)

$$\begin{array}{l} R_{f}^{m}(\eta )=f_{m-1}^{\prime \prime \prime }(\eta )+\sum_{k=0}^{m-1}\left(f_{m-1-k}f_{k}^{\prime \prime }-f_{m-1-k}^{\prime }f_{k}^{\prime }\right)+\beta_{1}\sum_{k=0}^{m-1}f_{m-1-k}\left[\sum_{l=0}^{k}\left(2f_{k-1}^{\prime }f_{l}^{\prime \prime }-f_{k-1}f_{l}^{\prime \prime \prime }\right)\right]\\ \;+\beta_{2}\sum_{k=0}^{m-1}\left(f_{m-1-k}^{\prime \prime }f_{k}^{\prime \prime }-f_{m-1-k}f_{k}^{iv}\right)+\lambda \left(\theta_{m-1}+N\phi_{m-1}\right), \end{array}$$ (48)

$$R_{\theta }^{m}(\eta )=\left(1+Rd\right)\theta_{m-1}^{\prime \prime }(\eta )+\text{Pr}\sum_{k=0}^{m-1}(f_{m-1-k}\theta_{k}^{\prime }-2f_{m-1-k}^{\prime }\theta_{k})-2\text{Pr}\varepsilon_{1}f_{m-1}^{\prime },$$ (49)

$$R_{\phi }^{m}(\eta )=\phi_{m-1}^{\prime \prime }(\eta )+Sc\overset{m-1}{\underset{k=0}{\sum }}(f_{m-1-k}\phi_{k}^{\prime }-2f_{m-1-k}^{\prime }\phi_{k})-Sc\gamma \phi_{m-1}-2Sc\varepsilon_{21}f_{m-1}^{\prime },$$ (50)

$$\chi_{m}=\{\begin{array}{c} 0,\;m\leq 1,\\ 1,\;m>1. \end{array}$$ (51)

The mth-order deformation problems have the solutions

$$f_{m}(\eta )=f_{m}^{*}(\eta )+C_{1}+C_{2}e^{\eta }+C_{3}e^{-\eta },$$ (52)

$$\theta_{m}(\eta )=\theta_{m}^{*}(\eta )+C_{4}e^{\eta }+C_{5}e^{-\eta },$$ (53)

$$\phi_{m}(\eta )=\phi_{m}^{*}(\eta )+C_{6}e^{\eta }+C_{7}e^{-\eta }.$$ (54)

in which $f_{m}^{*}(\eta ),$ $g_{m}^{*}(\eta ),$ $\theta_{m}^{*}(\eta )$ and $\phi_{m}^{*}(\eta )$ denote the special solutions.

## Convergence Analysis

**Fig 1:**
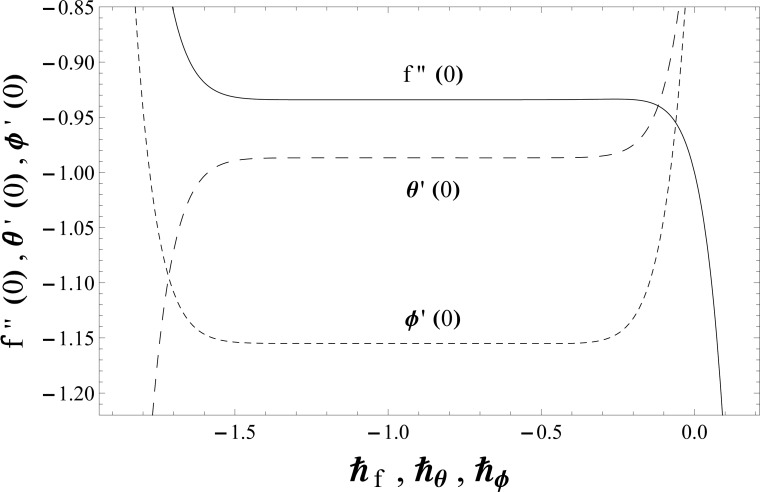
The ℏ− curves for the functions *f*(*η*), *θ*(*η*) and *ϕ*(*η*) when *β*_1_ = *β*_2_ = 0.2 = *γ* = *Rd*, *λ* = 0.1 = *N*, Pr = 1.0 = *Sc* and *ε*_1_ = 0.3 = *ε*_2_.

The series solutions (40)–(42) involve the auxiliary parameters $\hbar_{f},$ $\hbar_{\theta }$ and $\hbar_{\phi }.$ These parameters are useful in adjusting and controlling the convergence of the obtained series solutions. The proper values of these parameters are quite essential to construct the convergent solutions via homotopy analysis method. To choose the suitable values of $\hbar_{f},$ $\hbar_{\theta }$ and $\hbar_{\phi },$ the ℏ−curves are plotted at 13th order of approximations. Fig 1 clearly depicts that the convergence region lies within the domain $-1.40\leq \hbar_{f}\leq -0.35,$ $-1.45\leq \hbar_{\theta }\leq -0.35$ and $-1.45\leq \hbar_{\phi }\leq -0.40.$ Furthermore the series solutions converge in the whole region of *η* when $\hbar_{f}=-1.0=\hbar_{\theta }=\hbar_{\phi }.$ Table 1 shows that the 11th order of approximations are sufficient for the convergent series solutions.

**Table 1:** Convergence of HAM solutions for different order of approximations when *β*_1_ = *β*_2_ = 0.2 = *γ* = *Rd*, *λ* = 0.1 = *N*, Pr = 1.0 = *Sc*, *ε*_1_ = 0.3 = *ε*_2_ and $\hbar_{f}=-1.0=\hbar_{\theta }=\hbar_{\phi }.$

Order of approximations	−*f*′′ (0)	−*θ*′ (0)	−*ϕ*′ (0)
1	0.91150	1.01333	1.15333
5	0.93405	0.98685	1.15531
11	0.93419	0.98680	1.15505
20	0.93419	0.98680	1.15505
35	0.93419	0.98680	1.15505
50	0.93419	0.98680	1.15505

## Results and Discussion

**Fig 2:**
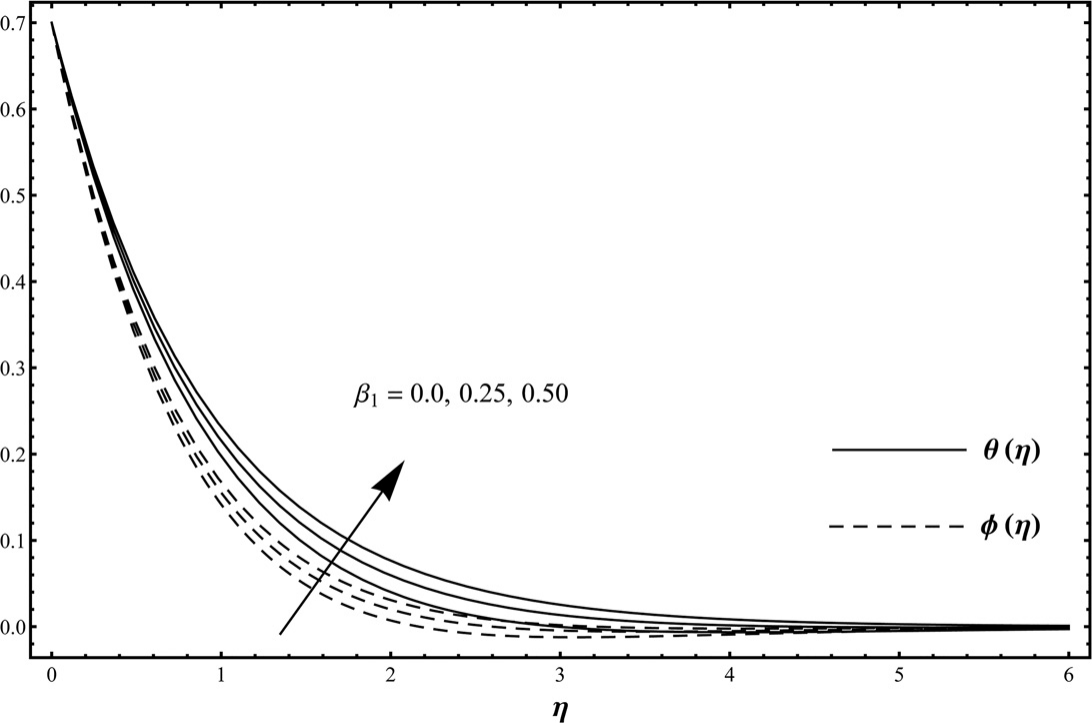
Temperature distribution function *θ*(*η*) and concentration distribution function *ϕ*(*η*) when *β*_1_ = 0.0, 0.25, 0.50 and *β*_2_ = 0.2 = *γ* = *Rd*, *λ* = 0.1 = *N*, Pr = 1.0 = *Sc*, *ε*_1_ = 0.3 = *ε*_2_.

**Fig 3:**
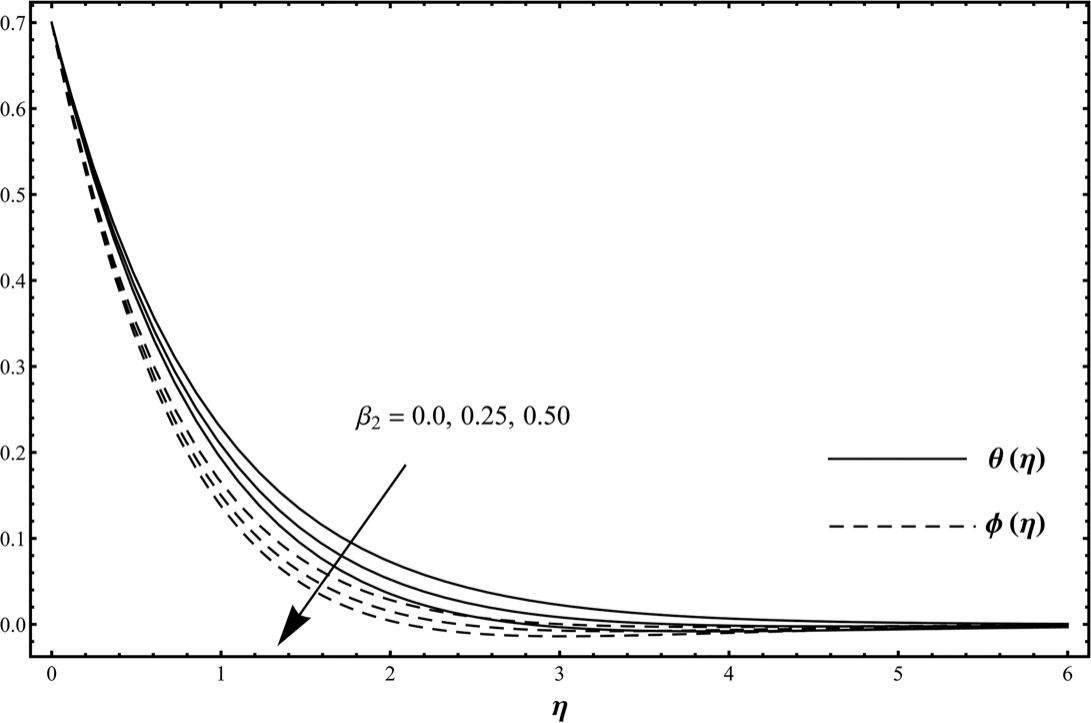
Temperature distribution function *θ*(*η*) and concentration distribution function *ϕ*(*η*) when *β*_2_ = 0.0, 0.25, 0.50 and *β*_1_ = 0.2 = *γ* = *Rd*, *λ* = 0.1 = *N*, Pr = 1.0 = *Sc*, *ε*_1_ = 0.3 = *ε*_2_.

**Fig 4:**
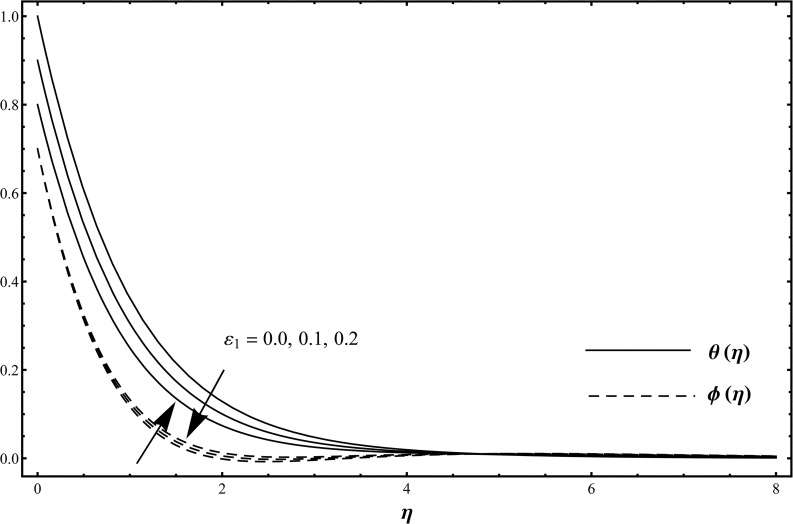
Temperature distribution function *θ*(*η*) and concentration distribution function *ϕ*(*η*) when *ε*_1_ = 0.0, 0.1, 0.2 and *β*_1_ = *β*_2_ = 0.2 = *γ* = *Rd*, *λ* = 0.1 = *N*, Pr = 1.0 = *Sc*, *ε*_2_ = 0.3.

**Fig 5:**
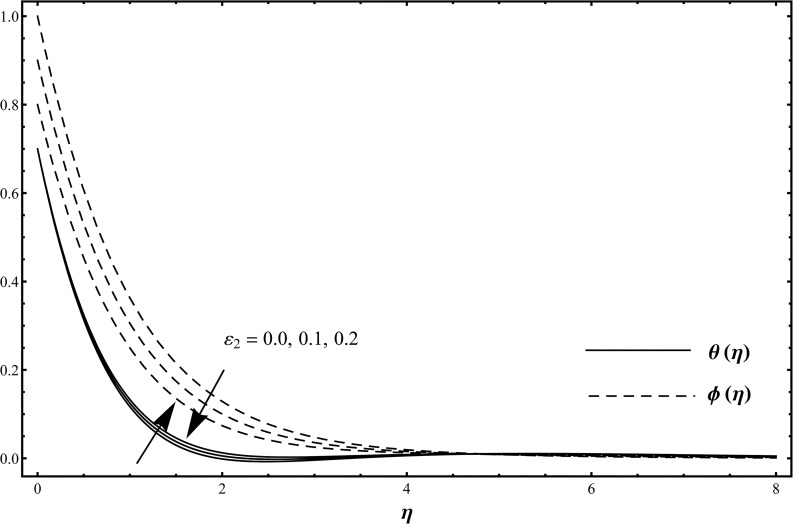
Temperature distribution function *θ*(*η*) and concentration distribution function *ϕ*(*η*) when *ε*_2_ = 0.0, 0.1, 0.2 and *β*_1_ = *β*_2_ = 0.2 = *γ* = *Rd*, *λ* = 0.1 = *N*, Pr = 1.0 = *Sc* and *ε*_1_ = 0.3.

**Fig 6:**
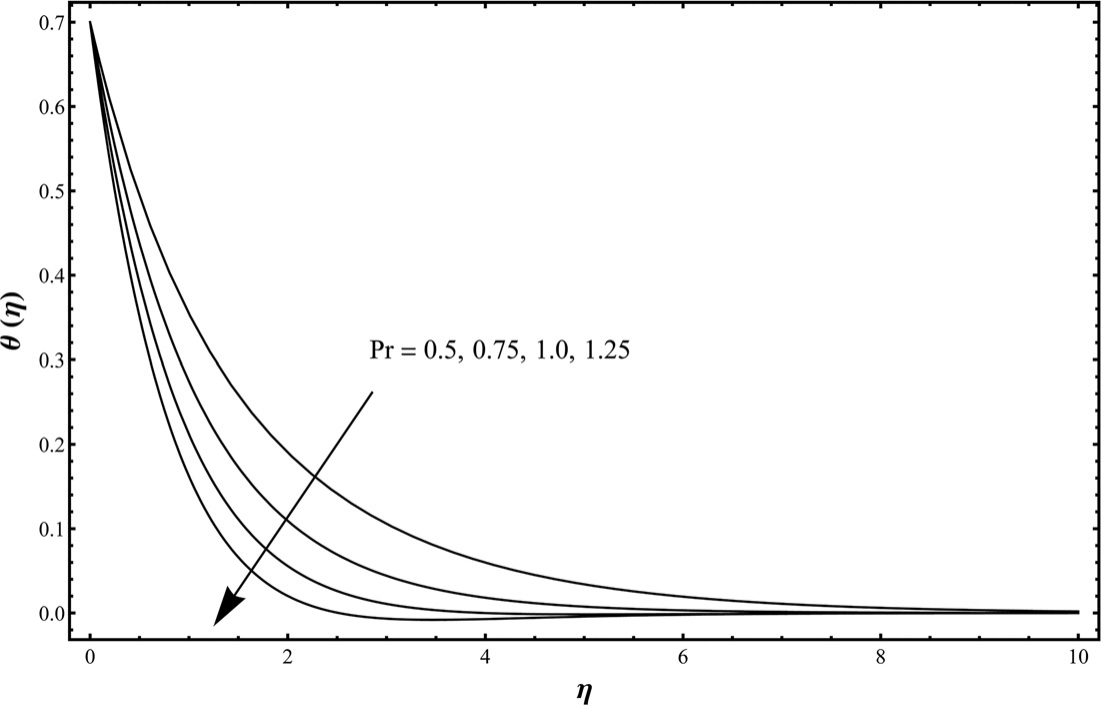
Temperature distribution function *θ*(*η*) when Pr = 0.5, 0.75, 1.0, 1.25 and *β*_1_ = *β*_2_ = 0.2 = *γ* = *Rd*, *λ* = 0.1 = *N*, *Sc* = 1.0, *ε*_1_ = 0.3 = *ε*_2_.

**Fig 7:**
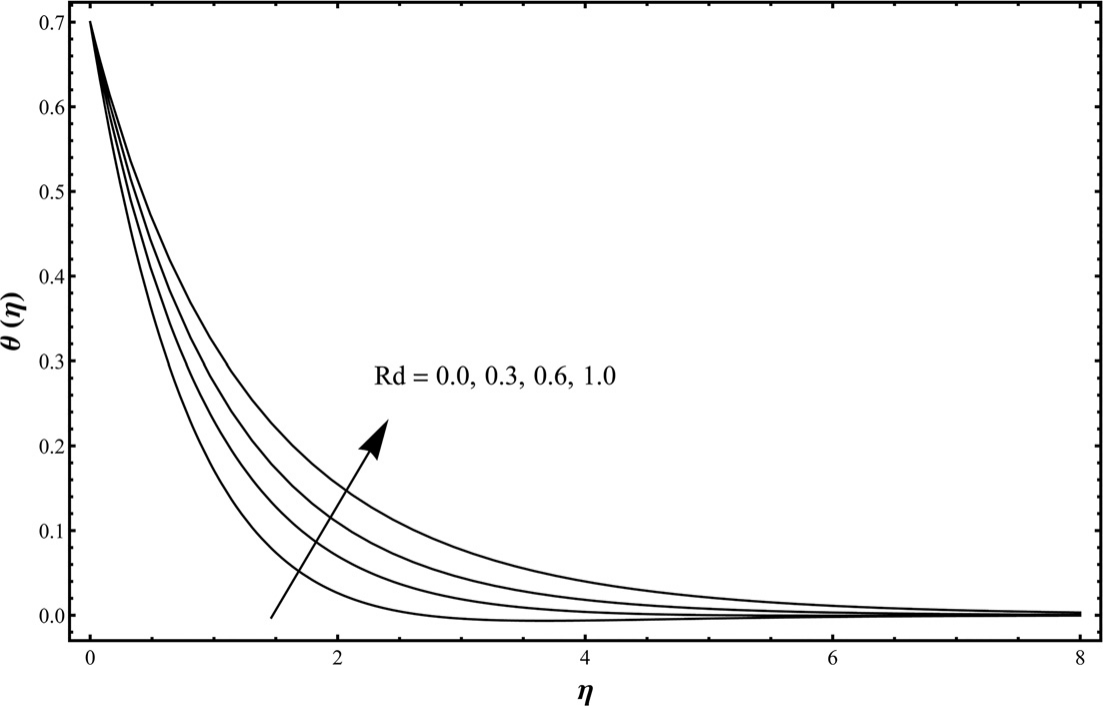
Temperature distribution function *θ*(*η*) when *Rd* = 0.0, 0.3, 0.6, 1.0 and *β*_1_ = *β*_2_ = 0.2 = *γ*, *λ* = 0.1 = *N*, Pr = 1.0 = *Sc*, *ε*_1_ = 0.3 = *ε*_2_.

**Fig 8:**
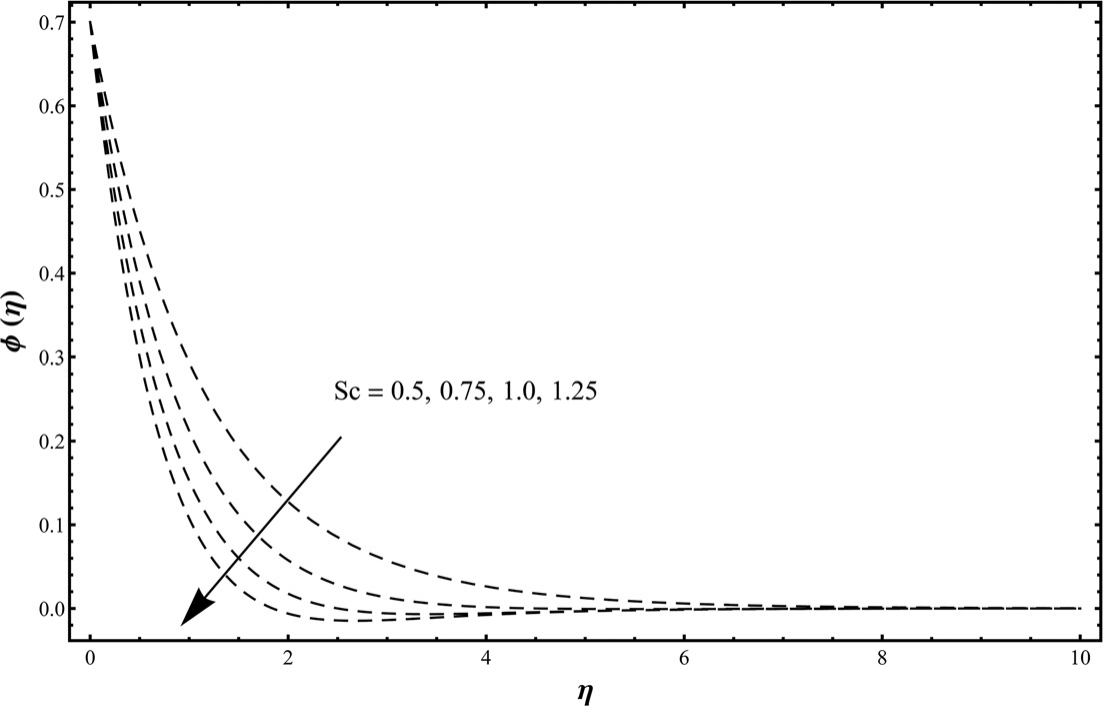
Concentration distribution function *ϕ*(*η*) when *Sc* = 0.5, 0.75, 1.0, 1.25 and *β*_1_ = *β*_2_ = 0.2 = *γ* = *Rd*, *λ* = 0.1 = *N*, Pr = 1.0, *ε*_1_ = 0.3 = *ε*_2_.

**Fig 9:**
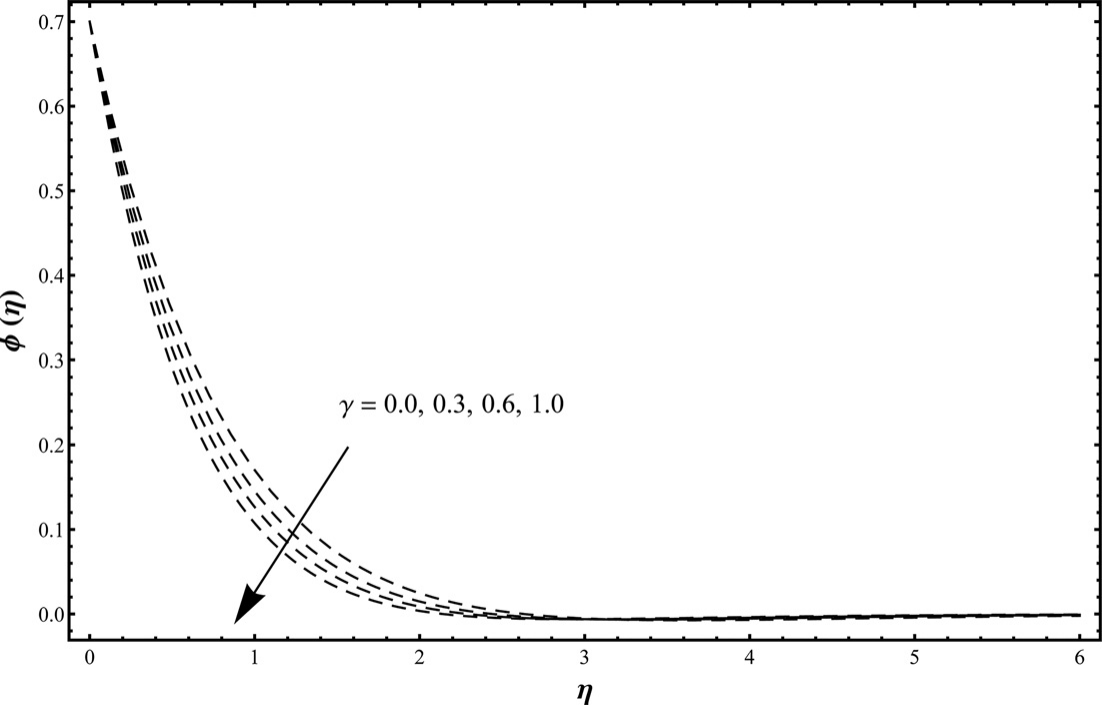
Concentration distribution function *ϕ*(*η*) when *γ* = 0.0, 0.3, 0.6, 1.0 and *β*_1_ = *β*_2_ = 0.2 = *Rd*, *λ* = 0.1 = *N*, Pr = 1.0 = *Sc*, *ε*_1_ = 0.3 = *ε*_2_.

This section presents the impacts of various emerging parameters including Deborah number in terms of relaxation time *β*_1_, Deborah number in terms of retardation time *β*_2_, Prandtl number Pr, thermal radiation parameter *Rd*, Schmidt number *Sc*, chemical reaction parameter *γ*, thermal stratification parameter *ε*_1_ and solutal stratification parameter *ε*_2_ on the dimensionless temperature profile *θ*(*η*) and concentration profile *ϕ*(*η*). This purpose is achieved through the plots in the Figs 2–9. Fig 2 is plotted to examine the effects of Deborah number *β*_1_ on the temperature profile *θ*(*η*) and concentration profile *ϕ*(*η*) when *β*_1_ = 0.0, 0.25, 0.50 and *β*_2_ = 0.2 = *γ* = *Rd*, *λ* = 0.1 = *N*, Pr = 1.0 = *Sc*, *ε*_1_ = 0.3 = *ε*_2_. Fig 2 examined that the temperature profile *θ*(*η*) and concentration profile *ϕ*(*η*) are enhanced when we use the larger values of Deborah number *β*_1_. Since Deborah number *β*_1_ has dependence on the relaxation time [4]. Larger values of Deborah number *β*_1_ implies to higher relaxation time. It is well known fact that the larger relaxation time fluids have higher temperature and concentration and smaller relaxation time fluids possess lower temperature and concentration. In view of this argument, both temperature profile *θ*(*η*) and concentration profile *ϕ*(*η*) are enhanced via larger Deborah number *β*_1_. The influence of Deborah number *β*_2_ on the dimensionless temperature and concentration fields when *β*_2_ = 0.0, 0.25, 0.50 and *β*_1_ = 0.2 = *γ* = *Rd*, *λ* = 0.1 = *N*, Pr = 1.0 = *Sc*, *ε*_1_ = 0.3 = *ε*_2_ is studied in Fig 3. Fig 3 clearly depicts that the temperature *θ*(*η*) and concentration *ϕ*(*η*) are decreasing functions of Deborah number *β*_2_ [11]. Here the Deborah number *β*_2_ is dependent on the retardation time. When we increase the values of Deborah number *β*_2_, the retardation time is increased. Such increase in retardation time is responsible for the reduction in the temperature *θ*(*η*) and concentration *ϕ*(*η*). Here it is interesting to mention that *β*_1_ = 0 = *β*_2_ correspond to viscous fluid case and *β*_2_ = 0 shows the Maxwellian fluid flow situation. From experimental point of view, it is quite obvious that the values of *β*_2_ are not much than the values of *β*_1_. Influence of thermal stratification parameter *ε*_1_ on the temperature *θ*(*η*) and concentration *ϕ*(*η*) is shown in Fig 4 when *ε*_1_ = 0.0, 0.1, 0.2 and *β*_1_ = *β*_2_ = 0.2 = *γ* = *Rd*, *λ* = 0.1 = *N*, Pr = 1.0 = *Sc*, *ε*_2_ = 0.3. Here the temperature and thermal boundary layer thickness are decreased while concentration and its related boundary layer thickness are increased when we increase in thermal stratification parameter. When the thermal stratification effect is taken into account, the effective temperature difference between the surface and the ambient fluid is decreased while opposite behavior is observed for concentration profile [28]. Influence of solutal stratification parameter *ε*_2_ on the temperature profile *θ*(*η*) and concentration profile *ϕ*(*η*) is shown in Fig 5 when *ε*_2_ = 0.0, 0.1, 0.2 and *β*_1_ = *β*_2_ = 0.2 = *γ* = *Rd*, *λ* = 0.1 = *N*, Pr = 1.0 = *Sc* and *ε*_1_ = 0.3. The temperature profile is enhanced while the concentration profile is reduced with an increase in solutal stratification parameter [29]. Influence of Prandtl number on the temperature profile is shown in Fig 6 when Pr = 0.5, 0.75, 1.0, 1.25 and *β*_1_ = *β*_2_ = 0.2 = *γ* = *Rd*, *λ* = 0.1 = *N*, *Sc* = 1.0, *ε*_1_ = 0.3 = *ε*_2_. The temperature and thermal layer thickness are reduced for the increasing values of Prandtl number. Physically larger Prandtl fluids possess lower thermal diffusivity and smaller Prandtl fluids have higher thermal diffusivity. This change in thermal diffusivity causes a reduction in the temperature and thermal boundary layer thickness. Basically Prandtl number is the ratio of momentum diffusivity to thermal diffusivity. In heat transfer, Prandtl number is used to control the thicknesses of momentum and thermal boundary layers. Fig 7 is plotted to examine the change in temperature profile when *Rd* = 0.0, 0.3, 0.6, 1.0 and *β*_1_ = *β*_2_ = 0.2 = *γ*, *λ* = 0.1 = *N*, Pr = 1.0 = *Sc*, *ε*_1_ = 0.3 = *ε*_2_. Fig 7 describes that the temperature and thermal boundary layer thickness are enhanced with an increase in the thermal radiation parameter. Larger values of thermal radiation parameter provide more heat to working fluid that shows an enhancement in the temperature and thermal boundary layer thickness [20]. Influence of Schmidt number on the concentration field is shown in Fig 8 when *Sc* = 0.5, 0.75, 1.0, 1.25 and *β*_1_ = *β*_2_ = 0.2 = *γ* = *Rd*, *λ* = 0.1 = *N*, Pr = 1.0, *ε*_1_ = 0.3 = *ε*_2_. It is clearly observed that the concentration and its related boundary layer thickness are decreasing functions of Schmidt number. Schmidt number is inversely proportional to the diffusion coefficient. Hence an increase in Schmidt number corresponds to a smaller diffusion coefficient. Such smaller diffusion coefficient creates a reduction in the concentration field. Fig 9 is plotted to investigate the effects of chemical reaction parameter when *γ* = 0.0, 0.3, 0.6, 1.0 and *β*_1_ = *β*_2_ = 0.2 = *Rd*, *λ* = 0.1 = *N*, Pr = 1.0 = *Sc*, *ε*_1_ = 0.3 = *ε*_2_. It is noticed from Fig 9 that the concentration and its associated boundary layer thickness are decreasing functions of chemical reaction parameter. Chemical reaction increases the rate of interfacial mass transfer. The reaction reduces the local concentration, thus increasing the concentration gradient and its flux. As a result, concentration of the chemical species in the boundary layer decreases with an increase in chemical reaction parameter. Tables 2 and 3 show the numerical values of the local Nusselt and the local Sherwood numbers for different values of *β*_1_, *β*_2_, *λ*, *N*, Pr, *Sc*, *Rd*, *γ*, *ε*_1_ and *ε*_2_. The values of local Nusselt and the local Sherwood numbers are decreased by increasing *ε*_1_ and *ε*_2_ while these values are increased for the larger *λ* and *N*. Table 4 is computed to validate the present results with the previous published results in a limiting sense. Here we compared our results for a Maxwell fluid case. From this Table, we examined that the present series solutions have good agreement with the numerical solutions of Megahed [42] in limiting sense.

**Table 2:** Values of local Nusselt number (Re_*x*_)^−1/2^ *Nu*_*x*_ and local Sherwood number (Re_*x*_)^−1/2^ *Sh*_*x*_ for different values of *β*_1_, *β*_2_, *λ* and *N* when Pr = 1.0 = *Sc*, *Rd* = 0.2 = *γ* and *ε*_1_ = 0.3 = *ε*_2_.

*β*_1_	*β*_2_	*λ*	*N*	−(1 + *Rd*)*θ*′ (0)	−*ϕ*′ (0)
0.0	0.2	0.1	0.1	0.97961	0.97656
0.2				0.95671	0.96073
0.4				0.93563	0.94621
0.2	0.0	0.1	0.1	0.92966	0.94168
	0.2			0.95671	0.96073
	0.4			0.97899	0.97648
0.2	0.2	0.0	0.1	0.95032	0.95590
		0.2		0.96267	0.96528
		0.5		0.97862	0.97761
0.2	0.2	0.1	0.0	0.95636	0.96045
			0.2	0.95706	0.96101
			0.5	0.95809	0.96186

**Table 3:** Values of local Nusselt number (Re_*x*_)^−1/2^ *Nu*_*x*_ and local Sherwood number (Re_*x*_)^−1/2^ *Sh*_*x*_ for different values of Pr, *Sc*, *Rd*, *γ*, *ε*_1_ and *ε*_2_ when *β*_1_ = 0.2 = *β*_2_ and *λ* = 0.1 = *N*.

*Pr*	*Sc*	*Rd*	*γ*	*ε*_1_	*ε*_2_	−(1 + *Rd*)*θ*′ (0)	−*ϕ*′ (0)
0.5	1.0	0.2	0.2	0.3	0.3	0.60210	0.96913
1.0						0.95671	0.96073
1.5						1.24219	0.95807
1.0	0.5	0.2	0.2	0.3	0.3	0.95754	0.61970
	1.0					0.95671	0.96073
	1.5					0.95647	1.22937
1.0	1.0	0.0	0.2	0.3	0.3	0.89755	0.95936
		0.3				0.98338	0.96143
		0.5				1.03215	0.96284
1.0	1.0	0.2	0.0	0.3	0.3	0.95674	0.89936
			0.2			0.95671	0.96073
			0.5			0.95667	1.04369
1.0	1.0	0.2	0.2	0.0	0.3	1.08817	0.96706
				0.5		0.86751	0.95648
				1.0		0.63912	0.94576
1.0	1.0	0.2	0.2	0.3	0.0	0.95736	1.12364
					0.5	0.95628	0.85206
					1.0	0.95519	0.58011

**Table 4:** Comparative values of –*f*″(0) for various values of *β*_1_ when *β*_2_ = *λ* = *N* = 0.

*β*_1_	Megahed [42]	Present work
0.0	0.999978	1.0000
0.2	1.051945	1.0519
0.4	1.101848	1.1019
0.6	1.150160	1.1501
0.8	1.196690	1.1967
1.2	1.285253	1.2853
1.6	1.368641	1.3686
2.0	1.447616	1.4476

## Conclusions

Influence of double stratification in mixed convection flow of an Oldroyd-B fluid with thermal radiation and chemical reaction are examined. This analysis reduces to the Maxwell fluid flow case when *β*_2_ = *0*. The main findings of this research are given below.

- Temperature and concentration fields are increased when we increase the values of *β*_1_. Here the relaxation time is enhanced when we give rise to Deborah number that leads to the higher temperature and concentration fields. This observation is similar that obtained in [18].
- Temperature and thermal boundary layer thickness are decreased when Prandtl number increases. Prandtl number is used to control the heat transfer rate in industrial process [29]. The proper value of Prandtl number is quite essential to control the heat transfer rate in industrial and engineering processes.
- Temperature and thermal boundary layer thickness are increasing functions of thermal radiation parameter. An increase in thermal radiation parameter provides more heat to fluid due to which larger temperature and thicker thermal boundary layer thickness are achieved.
- Influence of thermal stratification parameter on the temperature and concentration fields are opposite [30]. Here temperature is decreased while concentration is enhanced for the higher values of thermal stratification parameter.
- Temperature and concentration fields show opposite behavior for solutal stratification parameter. It is also observed that the thermal and concentration boundary layer thicknesses are reverse for the larger solutal stratification parameter.
- Concentration field and associated boundary layer thickness are reduced when we increase the values of chemical reaction parameter. For *γ* = 0 our analysis reduces to the case when there is no chemical reaction.
- Table 4 shows that our solutions have an excellent agreement with the previous published numerical results in limiting sense.
- The used technique for the solutions development and analysis has advantages over the other in the sense of following points:
  - It is independent of small/large physical parameters.
  - It provides a simple way to ensure the convergence of series solutions.
  - It provides a large freedom to choose the base functions and related auxiliary linear operators.
- Besides this the presented analysis is capable of describing relaxation and retardation times feature which many polymers show. Such analysis is particularly useful in polymer extrusion coating process, blood related viscoelastic effects in hemodynamic [43–45] etc. Such analysis provides a stimulus for future investigations on the topic in regimes of magnetohydrodynamics and convective conditions of heat transfer at the surface. It should be pointed out that presented analysis is not able to describe the rheological fluid properties in terms of normal stress effects, shear thinning and shear thickening features. Further the present analysis just deals with the hydrodynamic flow situation due to which the effects of viscous dissipation and Joule heating are ignored. In future one can discuss the hydromagnetic flow case in the presence of Joule heating and viscous dissipation.

## Appendix

Here we include the derivation of the governing equation. The constitutive equation for an Oldroyd-B fluid is expressed as follows:

$$\tau^{ij}=-p\delta^{ij}+S^{ij},$$ (A1)

where **τ** ^*ij*^ represents the components of Cauchy stress tensor, *p* the pressure, *δ* ^*ij*^ the components of identity tensor and the components of an extra stress tensor **S** ^*ij*^ is defined as follows:

$$\left(1+\lambda_{1}\frac{D}{Dt}\right)S^{ij}=\mu \left(1+\lambda_{2}\frac{D}{Dt}\right)A_{1}^{ij},$$ (A2)

where *μ* denotes the dynamic viscosity, *λ*_1_ and *λ*_2_ are the relaxation and retardation times respectively, $A_{1}^{ij}$ the components of first Rivlin-Ericksen tensor and $\frac{D}{Dt}$ the contravariant convective derivative which can be written below in the forms

$$\frac{Db^{i}}{Dt}=\frac{\partial b^{i}}{\partial t}+v^{r}b_{,r}^{i}-v_{,r}^{i}b^{r},$$ (A3)

$$\frac{Db^{ij}}{Dt}=\frac{\partial b^{ij}}{\partial t}+v^{r}b_{,r}^{ij}-v_{,r}^{i}b^{rj}-v_{,r}^{j}b^{ir}.$$ (A4)

The above expressions represent a contravariant vector and a contravariant tensor having rank 1 and 2 respectively, (where *v* ^*i*^ denote the components of velocity and “,” represents the covariant derivative). In case of Cartesian coordinates the covariant derivative reduces to the usual partial derivative. The equations governing the flow are

$$v_{,r}^{i}=0,$$ (A5)

$$\rho a=\tau_{,j}^{ij}-\rho g,$$ (A6)

where *ρ* denotes the fluid density, *g* the gravitational field and the definition of acceleration ‘*a*’ is

$$a^{i}=\frac{\partial v^{i}}{\partial t}+v^{r}v_{,r}^{i}.$$ (A7)

By applying the operator $\left(1+\lambda_{1}\frac{D}{Dt}\right),$ Eq (A6) gives

$$\rho \left(1+\lambda_{1}\frac{D}{Dt}\right)a^{i}=\left(1+\lambda_{1}\frac{D}{Dt}\right)\left(-\delta^{ij}p_{,j}+S_{,j}^{ij}\right)-\rho \left(1+\lambda_{1}\frac{D}{Dt}\right)g$$ (A8)

or

$$\rho \left(1+\lambda_{1}\frac{D}{Dt}\right)a^{i}=\left(1+\lambda_{1}\frac{D}{Dt}\right)\left(-\delta^{ij}p_{,j}\right)+{\left(\left(1+\lambda_{1}\frac{D}{Dt}\right)S^{ij}\right)}_{,j}-\rho \left(1+\lambda_{1}\frac{D}{Dt}\right)g.$$ (A9)

Assuming to derive the above equation that ${\left(\frac{D}{Dt}\right)}_{,j}=0$ and Eq (A2), we get the following equation:

$$\begin{array}{l} \;\rho \left(1+\lambda_{1}\frac{D}{Dt}\right)a^{i}\\ =\left(1+\lambda_{1}\frac{D}{Dt}\right)\left(-\delta^{ij}p_{,j}\right)+\mu {\left(\left(1+\lambda_{2}\frac{D}{Dt}\right)A_{1}^{ij}\right)}_{,j}-\rho \left(1+\lambda_{1}\frac{D}{Dt}\right)g\\ =\left(1+\lambda_{1}\frac{D}{Dt}\right)\left(-\delta^{ij}p_{,j}\right)+\mu \left(1+\lambda_{2}\frac{D}{Dt}\right)A_{1,j}^{ij}-\rho \left(1+\lambda_{1}\frac{D}{Dt}\right)g. \end{array}$$ (A10)

For an incompressible steady and two-dimensional flow one can write

$$\begin{array}{l} \rho a^{i}+\rho \lambda_{1}\frac{D}{Dt}a^{i}=-\delta^{ij}p_{,j}+\lambda_{1}\frac{D}{Dt}\left(-\delta^{ij}p_{,j}\right)\\ +\mu A_{1,j}^{ij}+\mu \lambda_{2}\frac{D}{Dt}A_{1,j}^{ij}-\rho g-\rho \lambda_{1}\frac{D}{Dt}g\;\text{for}\;i=1,2, \end{array}$$ (A11)

$$a^{1}=v^{r}v_{,r}^{1}=u\frac{\partial u}{\partial x}+v\frac{\partial u}{\partial y},$$ (A12)

$$a^{2}=v^{r}v_{,r}^{2}=u\frac{\partial v}{\partial x}+v\frac{\partial v}{\partial y},$$ (A13)

$$\delta^{ij}=\{\begin{array}{c} 1\;i=j\\ 0\;i\neq j \end{array},\;i,j=1,2.$$ (A14)

For *x*-component of momentum equation we have

$$\begin{array}{l} \frac{D}{Dt}a^{i}=v^{r}a_{,r}^{1}-v_{,r}^{1}a^{r}\\ \;=u\frac{\partial }{\partial x}\left(u\frac{\partial u}{\partial x}+v\frac{\partial u}{\partial y}\right)+v\frac{\partial }{\partial y}\left(u\frac{\partial u}{\partial x}+v\frac{\partial u}{\partial y}\right)\\ \;-\frac{\partial u}{\partial y}\left(u\frac{\partial v}{\partial x}+v\frac{\partial v}{\partial y}\right)-\frac{\partial u}{\partial x}\left(u\frac{\partial u}{\partial x}+v\frac{\partial u}{\partial y}\right)\\ \;=u^{2}\frac{\partial^{2}u}{\partial x^{2}}+v^{2}\frac{\partial^{2}u}{\partial y^{2}}+2uv\frac{\partial^{2}u}{\partial x\partial y} \end{array}$$ (A15)

$$\delta^{ij}p_{,j}=\frac{\partial p}{\partial x},$$ (A16)

$$\begin{array}{l} \frac{D}{Dt}\delta^{ij}p_{,j}=\frac{D}{Dt}\left(\delta^{11}p_{,1}+\delta^{12}p_{,2}\right)\\ \;=\frac{D}{Dt}\left(\frac{\partial p}{\partial x}\right),\\ \;=u\frac{\partial }{\partial x}\left(\frac{\partial p}{\partial x}\right)+v\frac{\partial }{\partial y}\left(\frac{\partial p}{\partial x}\right)-\frac{\partial u}{\partial x}\frac{\partial p}{\partial x}-\frac{\partial u}{\partial y}\frac{\partial p}{\partial x},\\ \;=u\frac{\partial^{2}p}{\partial x^{2}}+v\frac{\partial^{2}p}{\partial x\partial y}-\frac{\partial u}{\partial x}\frac{\partial p}{\partial x}-\frac{\partial u}{\partial y}\frac{\partial p}{\partial x}, \end{array}$$ (A17)

$$A_{1,j}^{ij}=A_{1,1}^{11}+A_{1,2}^{12}=\frac{\partial^{2}u}{\partial x^{2}}+\frac{\partial^{2}u}{\partial y^{2}},$$ (A18)

$$\begin{array}{l} \frac{D}{Dt}A_{1,j}^{ij}=u\frac{\partial^{3}u}{\partial x^{3}}+u\frac{\partial^{3}u}{\partial x\partial y^{2}}+v\frac{\partial^{3}u}{\partial x^{2}\partial y}+v\frac{\partial^{3}u}{\partial y^{3}}\\ \;-\left(u\frac{\partial^{2}v}{\partial x^{2}}+v\frac{\partial^{2}v}{\partial y^{2}}\right)\frac{\partial u}{\partial y}-\left(\frac{\partial^{2}u}{\partial x^{2}}+\frac{\partial^{2}u}{\partial y^{2}}\right)\frac{\partial u}{\partial x}. \end{array}$$ (A19)

Using Eqs (A12–A18) and Eq (A11) becomes

$$\begin{array}{l} \rho \left(u\frac{\partial u}{\partial x}+v\frac{\partial u}{\partial y}\right)\\ =-\rho \lambda_{1}\left(u^{2}\frac{\partial^{2}u}{\partial x^{2}}+2uv\frac{\partial^{2}u}{\partial x\partial y}+v^{2}\frac{\partial^{2}u}{\partial y^{2}}\right)-\frac{\partial p}{\partial x}\\ \;-\lambda_{1}\left(u\frac{\partial^{2}p}{\partial x^{2}}-\frac{\partial u}{\partial x}\frac{\partial p}{\partial x}-\frac{\partial u}{\partial y}\frac{\partial p}{\partial x}+v\frac{\partial^{2}p}{\partial x\partial y}\right)\\ \;+\mu \left(\frac{\partial^{2}u}{\partial x^{2}}+\frac{\partial^{2}u}{\partial y^{2}}\right)-\rho g\\ \;+\mu \lambda_{2}\left(\begin{array}{c} u\left(\frac{\partial^{3}u}{\partial x^{3}}+\frac{\partial^{3}u}{\partial x\partial y^{2}}\right)+v\left(\frac{\partial^{3}u}{\partial x^{2}\partial y}+\frac{\partial^{3}u}{\partial y^{3}}\right)\\ -\left(\frac{\partial^{2}u}{\partial x^{2}}+\frac{\partial^{2}u}{\partial y^{2}}\right)\frac{\partial u}{\partial x}-\left(\frac{\partial^{2}v}{\partial x^{2}}+\frac{\partial^{2}v}{\partial y^{2}}\right)\frac{\partial u}{\partial y} \end{array}\right). \end{array}$$ (A20)

Similarly *y*-component of momentum equation can be written as follows:

$$\begin{array}{l} \;\rho \left(u\frac{\partial v}{\partial x}+v\frac{\partial v}{\partial y}\right)\\ =-\rho \lambda_{1}\left(u^{2}\frac{\partial^{2}v}{\partial x^{2}}+2uv\frac{\partial^{2}v}{\partial x\partial y}+v^{2}\frac{\partial^{2}v}{\partial y^{2}}\right)-\frac{\partial p}{\partial y}\\ \;-\lambda_{1}\left(v\frac{\partial^{2}p}{\partial x^{2}}-\frac{\partial v}{\partial x}\frac{\partial p}{\partial x}-\frac{\partial v}{\partial y}\frac{\partial p}{\partial x}+u\frac{\partial^{2}p}{\partial x\partial y}\right)\\ \;+\mu \left(\frac{\partial^{2}v}{\partial x^{2}}+\frac{\partial^{2}v}{\partial y^{2}}\right)\\ \;+\mu \lambda_{2}\left(\begin{array}{c} u\left(\frac{\partial^{3}v}{\partial x^{3}}+\frac{\partial^{3}v}{\partial x\partial y^{2}}\right)+v\left(\frac{\partial^{3}v}{\partial x^{2}\partial y}+\frac{\partial^{3}v}{\partial y^{3}}\right)\\ -\left(\frac{\partial^{2}u}{\partial x^{2}}+\frac{\partial^{2}u}{\partial y^{2}}\right)\frac{\partial v}{\partial x}-\left(\frac{\partial^{2}v}{\partial x^{2}}+\frac{\partial^{2}v}{\partial y^{2}}\right)\frac{\partial v}{\partial y} \end{array}\right). \end{array}$$ (A21)

Here *u* and *v* show the velocities parallel to the *x*- and *y*-axes respectively and *ν* the kinematic viscosity.

Using the boundary layer approximations, i.e.,

$$\begin{array}{l} x=O\left(1\right),\;u=O\left(1\right),\;y=O\left(\delta \right),\;v=O\left(\delta \right),\\ \nu =\frac{\mu }{\rho }=O(\delta^{2}),\;\lambda_{1}=O(1),\;\lambda_{2}=O(1),p=O(1), \end{array}$$ (A22)

Eqs (A20) and (A21) reduce to the form

$$\begin{array}{l} \rho \left(u\frac{\partial u}{\partial x}+v\frac{\partial u}{\partial y}\right)+\rho \lambda_{1}\left(\begin{array}{c} u^{2}\frac{\partial^{2}u}{\partial x^{2}}+v^{2}\frac{\partial^{2}u}{\partial y^{2}}\\ +2uv\frac{\partial^{2}u}{\partial x\partial y} \end{array}\right)=-\frac{\partial p}{\partial x}+\mu \frac{\partial^{2}u}{\partial y^{2}}\\ \;+\mu \lambda_{2}\left(\begin{array}{c} u\frac{\partial^{3}u}{\partial x\partial y^{2}}+v\frac{\partial^{3}u}{\partial y^{3}}\\ -\frac{\partial u}{\partial x}\frac{\partial^{2}u}{\partial y^{2}}-\frac{\partial u}{\partial y}\frac{\partial^{2}v}{\partial y^{2}} \end{array}\right)-\rho g, \end{array}$$ (A23)

$$\frac{1}{\rho }\frac{\partial p}{\partial y}=-\frac{\lambda_{1}}{\rho }\left(-\frac{\partial v}{\partial y}\frac{\partial p}{\partial x}+u\frac{\partial^{2}p}{\partial x\partial y}\right).$$ (A24)

Using boundary condition (6), Eq (A23) becomes

$$-\frac{\partial p}{\partial x}=\rho_{\infty }g.$$ (A25)

Putting Eq (A25) in Eq (A23) we get

$$\rho \left(u\frac{\partial u}{\partial x}+v\frac{\partial u}{\partial y}\right)+\rho \lambda_{1}\left(\begin{array}{c} u^{2}\frac{\partial^{2}u}{\partial x^{2}}+v^{2}\frac{\partial^{2}u}{\partial y^{2}}\\ +2uv\frac{\partial^{2}u}{\partial x\partial y} \end{array}\right)=\mu \frac{\partial^{2}u}{\partial y^{2}}+\mu \lambda_{2}\left(\begin{array}{c} u\frac{\partial^{3}u}{\partial x\partial y^{2}}+v\frac{\partial^{3}u}{\partial y^{3}}\\ -\frac{\partial u}{\partial x}\frac{\partial^{2}u}{\partial y^{2}}-\frac{\partial u}{\partial y}\frac{\partial^{2}v}{\partial y^{2}} \end{array}\right)+\left(\rho_{\infty }-\rho \right)g.$$ (A26)

Taylor's series expansion about *ρ*_*∞*_ gives

$$\begin{array}{l} \rho =\rho_{\infty }+\left(\frac{\partial \rho }{\partial T}\right)\left(T-T_{\infty }\right)+\left(\frac{\partial \rho }{\partial C}\right)\left(C-C_{\infty }\right)\\ \;+\left(\frac{\partial^{2}\rho }{\partial T^{2}}\right)\frac{{\left(T-T_{\infty }\right)}^{2}}{2!}+\left(\frac{\partial^{2}\rho }{\partial C^{2}}\right)\frac{{\left(C-C_{\infty }\right)}^{2}}{2!}+.... \end{array}$$ (A27)

Neglecting square and higher terms of (*T* – *T*_*∞*_) and (*C* – *C*_*∞*_) we have

$$\left(\rho_{\infty }-\rho \right)=\beta_{T}\left(T-T_{\infty }\right)+\beta_{C}\left(C-C_{\infty }\right),$$ (A28)

This is Boussinesq approximation with

$$\beta_{T}=-\frac{1}{\rho }\left(\frac{\partial \rho }{\partial T}\right)\;\text{and}\;\beta_{C}=-\frac{1}{\rho }\left(\frac{\partial \rho }{\partial C}\right),$$ (A29)

Substituting Eq (A28) into Eq (A26) we get

$$\begin{array}{r} u\frac{\partial u}{\partial x}+v\frac{\partial u}{\partial y}+\lambda_{1}\left(\begin{array}{c} u^{2}\frac{\partial^{2}u}{\partial x^{2}}+v^{2}\frac{\partial^{2}u}{\partial y^{2}}\\ +2uv\frac{\partial^{2}u}{\partial x\partial y} \end{array}\right)=\nu \frac{\partial^{2}u}{\partial y^{2}}+\nu \lambda_{2}\left(\begin{array}{c} u\frac{\partial^{3}u}{\partial x\partial y^{2}}+v\frac{\partial^{3}u}{\partial y^{3}}\\ -\frac{\partial u}{\partial x}\frac{\partial^{2}u}{\partial y^{2}}-\frac{\partial u}{\partial y}\frac{\partial^{2}v}{\partial y^{2}} \end{array}\right)\;\\ +g\left(\beta_{T}\left(T-T_{\infty }\right)+\beta_{C}\left(C-C_{\infty }\right)\right). \end{array}$$ (A30)
